# The paradoxical relationship of sensorimotor deficit and lesion volume in acute ischemic stroke

**DOI:** 10.1093/jnen/nlaf046

**Published:** 2025-04-24

**Authors:** Réka Tóth, Nikoletta Szabó, Anna Törteli, Noémi Kovács, Ildikó Horváth, Krisztián Szigeti, Domokos Máthé, Tamás Zs Kincses, Ákos Menyhárt, Eszter Farkas

**Affiliations:** HCEMM-USZ Cerebral Blood Flow and Metabolism Research Group, HCEMM Nonprofit Ltd, Szeged, Hungary; Department of Cell Biology and Molecular Medicine, University of Szeged, Szeged, Hungary; Department of Radiology, University of Szeged, Szeged, Hungary; Department of Neurology, University of Szeged, Szeged, Hungary; HCEMM-USZ Cerebral Blood Flow and Metabolism Research Group, HCEMM Nonprofit Ltd, Szeged, Hungary; Department of Cell Biology and Molecular Medicine, University of Szeged, Szeged, Hungary; HCEMM-SU In Vivo Imaging Advanced Core Facility, Budapest, Hungary; Department of Biophysics and Radiation Biology, Faculty of Medicine, Semmelweis University, Budapest, Hungary; Department of Biophysics and Radiation Biology, Faculty of Medicine, Semmelweis University, Budapest, Hungary; HCEMM-SU In Vivo Imaging Advanced Core Facility, Budapest, Hungary; Department of Biophysics and Radiation Biology, Faculty of Medicine, Semmelweis University, Budapest, Hungary; Department of Radiology, University of Szeged, Szeged, Hungary; Department of Neurology, University of Szeged, Szeged, Hungary; HCEMM-USZ Cerebral Blood Flow and Metabolism Research Group, HCEMM Nonprofit Ltd, Szeged, Hungary; Department of Cell Biology and Molecular Medicine, University of Szeged, Szeged, Hungary; HCEMM-USZ Cerebral Blood Flow and Metabolism Research Group, HCEMM Nonprofit Ltd, Szeged, Hungary; Department of Cell Biology and Molecular Medicine, University of Szeged, Szeged, Hungary

**Keywords:** acute ischemic stroke, cerebral edema, lesion size, neuroimaging, neurological outcome

## Abstract

Understanding the relationship between the degree of neurological deficit and lesion volume is key to predicting outcomes in patients with acute ischemic stroke (AIS). Over the past 40 years, AIS research has relied on a perceived linear relationship between lesion volumes and neurological deficit. Here, we found that these variables do not show a relationship in a mouse model of AIS. Acute ischemic stroke was induced by transient (60 minutes) intraluminal microfilament occlusion of the middle cerebral artery in 15 male isoflurane (0.8%-1%)-anesthetized mice. Acute ischemic stroke-induced sensorimotor deficits were assessed daily for 72 hours using the Garcia Neuroscore Scale (GNS). Lesion size was estimated 72 hours after AIS using a rodent MRI system. Lesion sizes ranged from 17 to 130 mm^3^. In 3/15 mice (atypical cases: lesion <30 mm^3^ and GNS <11), small infarcts (14.6 ± 6.2 vs 51.7 ± 19.9 mm^3^, atypical vs typical) were associated with low GNS values at 72 hours (9 ± 2 vs 11 ± 2 pts; atypical vs typical). Consequently, we found no relationship between lesion size and GNS in this AIS model (*R* = 0.058). These results suggest that lesion size is not a reliable predictor of neurological outcome in AIS models.

## INTRODUCTION

Stroke is the third leading cause of death and the leading cause of disability worldwide, affecting 13.7 million people annually.^1^^,^^2^ By the mechanism of the disease, stroke is classified into ischemic and hemorrhagic subtypes. Acute ischemic stroke (AIS) accounts for 71% of all strokes globally and results from the transient or permanent obstruction of a cerebral artery.^1^^,^^2^ Occlusion of a vessel initiates a cascade of ischemic brain injury distal to the blockage, resulting in the evolution of a necrotized tissue, called a lesion. Brain lesion size is taken as a reliable indicator of neurological impairment and clinical outcomes in AIS patients. The routine assessment of neurological deficits is often predictive in AIS patients and helps clinicians to estimate the extent and location of lesions with confidence.^3^^,^^4^ Furthermore, the National Institutes of Health Stroke Scale score (NIHSS) is a widely accepted tool used in stroke centers to objectively measure stroke-related symptoms. The score increases with the severity of symptoms, with a score below 6 indicating mild stroke, 6-13 indicating moderate stroke, and over 14 indicating severe stroke.^5^ It correlates well with lesion size estimated through neuroimaging.^6^ The initial NIHSS score is the best predictor of long-term outcomes for AIS patients, according to clinical studies.^5^^,^^7^ Also, lesion sizes assessed within the first 72 hours of symptom onset are strong indicators of long-term recovery and final lesion volumes (LV).^8^ The widely used Alberta Stroke Programme Early CT Score (ASPECTS)^9^ also demonstrates a clear correlation between the extension of the lesion and poor functional outcome, predicting functional outcome based on diagnosed changes on neuroimaging.^10^^,^^11^

Convincing data support the correlation between the lesion size and neurological outcome. In a sufficiently homogeneous patient population with a large sample size, smaller lesion size in the anterior circulation territory was associated with a significantly better functional outcome.^12^ However, it is important to note that routine clinical prognosis may lead to incorrect conclusions in certain patients.^3^^,^^13^ Furthermore, there are studies with conflicting results, suggesting that predictions of outcome based solely on clinical symptoms may be inconsistent with lesion volume.^14^ In addition, deficit assessment in the subacute phase (at 6 or 7 days after AIS) has been suggested to have a stronger predictive value compared to acute deficits than baseline NIHSS scores. This suggests a possible progression of infarct volume and severity during the subacute phase.^15^ Taken together, further investigation is needed to resolve the conflicting attitudes surrounding this issue.

Over the past 2 decades, preclinical rodent models of AIS have often assumed a positive correlation between lesion size and neurological deficit.^16^ Many studies investigating pharmacological neuroprotection have used lesion size as their endpoint without examining neurological outcomes. However, this perspective along with other limitations (ie, disregard for age, sex, and comorbid factors), has misled the translational AIS research field, contributing to the so-called translational gap.^17^ The term “translational gap” stands for the inefficient translation of findings in rodents to humans, which raises questions among researchers.^17^ Pre-clinical models do not accurately represent all aspects of clinical states. Moreover, neurological deficits are not necessarily related to the size of the lesion in rodent stroke models.^17^ It should be noted that reducing the size of the lesion through pharmacological means may not always result in improved neurological outcomes.^18^ Yet, several studies have found it adequate to describe the neuroprotective potential of a treatment by measuring lesion size.^17^ The aim of this study is to investigate the relationship between lesion volume and the degree of early neurological deficits (within the first 3 days after AIS) and to identify any paradoxical relationships between the 2 variables.

## METHODS

### Animals

The study adhered to established ethical guidelines,^19^^,^^20^ and the experiments are reported in compliance with the ARRIVE guidelines.^21^ The experimental procedures were conducted in strict accordance with the guidelines of the National Food Chain Safety and Animal Health Directorate of Csongrád County, Hungary, and the guidelines of the Scientific Committee of Animal Experimentation of the Hungarian Academy of Sciences (updated Law and Regulations on Animal Protection: 40/2013. (II. 14.) Gov. of Hungary), following the EU Directive 2010/63/EU on the protection of experimental animals (Ref. nr. XXXII/4050/2020 and I-74-23/2022. MÁB).

The animals were housed under constant conditions of temperature (23 °C), humidity, and lighting (12:12 h light/dark cycle, lights on at 7 AM). Standard rodent chow and tap water were supplied ad libitum. Adult male 4–4.5-month-old C57BL/6 mice (Charles River Laboratories; weighing 26.83 ± 3.91 g; *n* = 20, from the husbandry of the Biological Research Centre, Szeged, Hungary) were used in this study. The animals were anesthetized with isoflurane (4% for induction, 0.6%-0.9% for maintenance in N_2_O:O_2_, 2:1) and allowed to breathe spontaneously through a nose cone. Body temperature was maintained at 37 °C by using a heating pad equipped with a temperature probe and blanket feedback-controlled system (CODA Monitor, Kent Scientific Corporation).

### Induction of AIS: modified microfilament occlusion of the middle cerebral artery

Focal cerebral ischemia was induced by occluding the left middle cerebral artery (MCAO) using the Koizumi method^22^ with in-house modifications to allow complete reperfusion. The mouse was placed in the supine position, and the skin of the neck was disinfected with ethanol. Lidocaine (1%) was administered at the incision site, which was made between the sternum and the chin. The left common carotid artery (CCA), carotid fork, external carotid artery (ECA), and internal carotid artery (ICA) were carefully separated. A Micro Serrefine (Fine Science Tools (USA), Inc.) was then placed on the CCA. A silk suture (Fine Science Tools) was gently looped around the ECA, and the 2 ends of the thread were tightly secured with a forceps to effectively obstruct blood flow in the ECA for approximately 60 minutes. A small incision was made in the CCA below the carotid bifurcation using a 30 G needle. A 230-µm diameter silicon-coated microfilament (Doccol Corp.) was then inserted through the incision and advanced into the CCA, the ICA, and finally to the middle cerebral artery (MCA) branch, which was identified by a sudden resistance. Successful MCAO was confirmed using a needle laser Doppler probe (Probe 403 connected to PeriFlux 5000; Perimed AB, Sweden) attached to the parietal bone. After 15 minutes of baseline, the experimental protocol included 60 minutes of MCAO followed by complete reperfusion induced by microfilament retrieval. Animals with an initial drop in cerebral blood flow (CBF) below 25% of the baseline perfusion were included in the study (CBF immediately following filament insertion: 13.11 ± 6.00%, mean ± SD; lowest CBF: 2.95 ± 1.35%, mean ± SD). Finally, the silk around the ECA was loosened, and the Micro Serrefine was carefully removed from the CCA. The wound was sutured, disinfected, and the non-steroidal anti-inflammatory agent carprofen was administered subcutaneously (5 mg kg^−1^) (Rycarfa, Tolnagro) in parallel with 0.5 ml saline (0.9% NaCl solution) for fluid replacement. The mice were then allowed to recover in an incubator cage at 30 °C for 60 minutes. Once fully conscious, the mice were returned to the animal facility and reunited with their cage mates.

### Post-operative care of mice after AIS

Postoperative care included the provision of food, water, and nutritional supplements as recommended by Lourbopoulos et al^23^ and Pinto et al.^24^ During the 24-72 hours after surgery, maximal nutritional support was provided by allowing ad libitum access to soaked pellet and jelly food in a Petri dish in the cage. Furthermore, food supplementation was complemented with twice-daily syringe feeding of jelly food. Fluid supplementation was achieved by subcutaneous administration of 0.3 ml of saline containing 5% glucose and 50%-50% Duphalyte (Zoetis Hungary Kft) twice daily. Carprofen was administered every 12 hours.

### Experimental protocol

**Figure 1.**
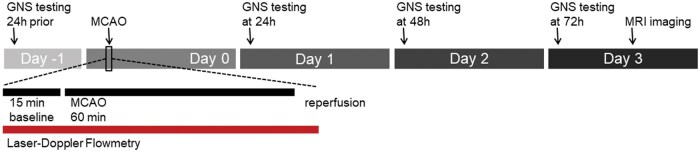
Experimental protocol. Animals were evaluated using the Garcia Neuroscore Scale (GNS) 1 day before the onset of ischemia (−24 h). On the day of surgery, unilateral middle cerebral artery occlusion (MCAO) was performed with simultaneous monitoring of CBF by laser Doppler flowmetry (LDF). The LDF acquisition protocol included 15 minutes of baseline, 60 minutes of MCAO, and an additional 5 minutes of acquisition during complete reperfusion. Daily GNS testing was performed at 24, 48, and 72 hours after acute ischemic stroke, with MRI performed on day 3.

On day −1, animals underwent neurological examination (Garcia Neuroscore Scale scoring) to assess baseline values. On day 0, MCAO surgery was performed, and the animals were allowed to recover for 24 hours. After 24 hours of recovery, neurological deficits due to AIS were assessed every 24 hours until day 3 post surgery. On day 3, after 72 hours of survival time, MRI was performed to determine infarct volume. In 5 representative animals, laser Doppler flowmetry was used to measure perfusion changes during the experimental protocol as indicated (Figure 1).

### Characterization of post-AIS neurological deficit

**Figure 2.**
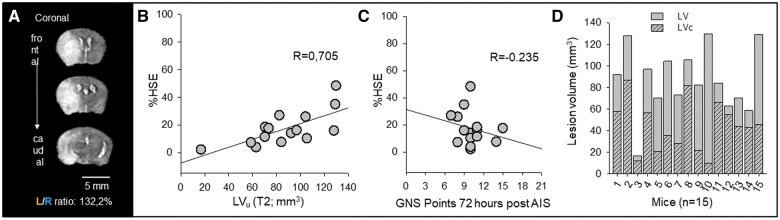
Correcting for edema in MRI images accurately estimates lesion volume; Garcia Neuroscore Scale (GNS) values do not correspond to the corrected LV. (A) The left/right hemispheric ratio (L/R ratio) was used to estimate the swelling of the left hemisphere affected by acute ischemic stroke (AIS). Note the shift of the midline and the expansion of the left hemisphere. (B) Tissue swelling or edema (increase in hemispheric volume due to space-occupying edema, %HSE) shows a linear relationship with lesion volume (LV) on T2 weighted MRI images (linear regression: *R* = 0.705, *P* = .003, *P* < 0.05). (C) Tissue swelling in MRI images shows no correlation with the GNS values measured 72 hours post-AIS (linear regression: *R* = 0.235, *P* = .398). (D) Edema correction provides a better estimation of true lesion volume (corrected lesion volume, LVc).

Animals underwent neurological testing 24 hours prior to surgery, and daily from 24 to 72 hours after MCAO (Figure 2). The Garcia Neuroscore Scale, a scoring system specifically designed to evaluate sensorimotor deficits following ischemic brain injury in rodents was utilized.^25^^,^^26^ The GNS score ranges from 0 (severe deficit) to 21 (no deficit). All test domains were performed in the same order for each animal with 3 investigators present at a time to ensure unbiased scoring.

### Evaluation of ischemic lesions by MRI

MRI scanning was performed at 72 hours survival using a Mediso NanoScan PET/MRI 3 T system (Mediso Ltd) equipped with a maximum of 450 mT m^−1^ gradients (Figure 2). A dedicated mouse head volume coil (inner diameter 30 mm) was used for both transmission and reception. The animals were placed on a heated pallet during scanning, and 1%-2% isoflurane was used for anesthesia. Respiration rate and the temperature were continuously monitored, and isoflurane concentration was adjusted accordingly. The T2-weighted fast spin echo scan was acquired with a three-dimensional acquisition scheme and the following parameters: 35 × 35 mm field of view, 220 × 220 acquisition matrix size, and 96 adjacent slices of 0.2-mm slice thickness. The echo-train length was set to 96, the echo time was 69 ms, and the repetition time was 2 s with one average to deliver a total acquisition time of ∼6.5 minutes. The diffusion-weighted scan consisted of 2 *b* values (*b* = 0 and 800 s mm^−2^, δ  =  2.4 ms, Δ  =  12 ms) along 3 perpendicular directions, each repeated 14 times. The study utilized the spin-echo echo-planar imaging pulse sequence to produce high-quality images of 22 adjacent slices (0.7-mm thickness) within a total acquisition time of 10 minutes. The field of view was set at 25 × 25 mm, and the acquisition matrix was optimized at 80 × 80. The echo time was set at 59.1 milliseconds using 300-kHz bandwidth and a repetition time of 5 seconds. To ensure accuracy, susceptibility and eddy current distortion corrections were applied during the reconstruction based on reference echo scans. Apparent diffusion coefficient (ADC) values were calculated for the lesion, the whole ipsilateral hemisphere to stroke, and the whole contralateral intact hemisphere using InterView Fusion software (Mediso). ADC values were calculated for the lesion, the whole ipsilateral hemisphere to stroke, and the whole contralateral intact hemisphere using InterView Fusion software (Mediso). Hemispheric volumes (HV) and LV were calculated from the T2FSE sequences and ADC maps.

### Data analysis and statistics

The mice were coded independently and randomized. Three investigators who were blinded to the analysis performed the surgeries, neuroscoring, MRI imaging, and infarct size calculation. Volumetric data were used to calculate the ratio of left/right brain hemispheres (L/R ratio) and hemispheric LV (%HLV). To correct for the lesion-expanding effect of tissue edema, the method validated by Gerriets et al^27^ was used. Edema-corrected LV (LVc) for lesions measured on T2 sequences were compared to LV uncorrected for edema. The space-occupying effect of edema formation was expressed using the increase of total hemispheric volume (%HSE). Data are presented as mean ± SD. Parametric or non-parametric statistics were chosen based on the results of a Shapiro-Wilk test of normality performed on the data sets. The statistical analysis was conducted using SigmaPlot 12.5 (Systat Software, Inc.). The specific statistical methods used are described in detail in each figure legend.

## RESULTS

### Ranging of T2-weighted LV and garcia neuroscore scale points

Our AIS model had a mortality rate of 25%, which is consistent with earlier data from the relevant literature reporting mortality rates of up to 50% following MCAO in mice^28^. Five out of 20 animals did not survive to be enrolled for MRI. The LV ranged between 17 and 130 mm^3^, corresponding to a relative size of 7.31%-54.17% of the total left cerebral hemisphere (hemispheric lesion volume, HLV%; 35.71 ± 12.06%). The regions of T2-weighted hyperintensity were consistently accompanied by areas of restricted diffusion, as measured in ADC maps (5.01 ± 0.32 × 10^−4^ vs 7.89 ± 0.60 × 10^−4^ mm^2^ seconds^−1^; lesioned vs intact hemisphere). This confirmed the development of the lesion. The volumes of the areas with decreased ADC were always within the T2 lesion area and ranged between 3.0 and 115.6 mm^3^ and were smaller than the adherent LV (range between 1.29 and 46.55 HLV%; mean ± SD 23.87 ± 11.94 HLV%). The GNS scores ranged from 7 to 15 at 72 hours of survival.

The animals were divided into 3 subgroups based on LVs: mild injury (<80 mm^3^), moderate injury (80-100 mm^3^), and severe injury (>100 mm^3^) (Figure S1). Garcia Neuroscore Scale values remained consistent over time within each group (*f*_time_ = 0.176, *p*_time_ = 0.684). The GNS score scattered the most in the Mild lesion group, and did not show significant differences between the mild, moderate, and severe lesion groups at any time point during the survival period (*f*_group_ = 0.815, *p*_group_ = 0.470). Therefore, neurological deficits could not be directly linked to LVs.

### Progression of cerebral edema compromises LV estimation

Cerebral edema progression significantly affected the accurate estimation of hemispheric LVs. Differences in total hemispheric volumes were observed due to brain swelling, as indicated by the left-right hemispheric ratio (L/R ratio), which ranged between 102.2% and 165.4%; with a mean of 121.90 ± 16.94% (Figure 2A). Although there was no correlation between L/R ratio and GNS at 72 hours (*R* = 0.242, *P* = .384), the increase in volume of the left hemisphere distorted the estimation of LVs (*R* = 0.724, *P* = .0023). The space-occupying effect of brain edema was quantified by calculating the increase in volume of the affected hemisphere (%HSE). %HSE is a numerical expression of the measured L/R ratio values, displaying a linear relationship between the 2 values (*R* = 0.997, *P* = .009). The swollen tissue within and around the lesion linearly increased the volume of the affected hemisphere by 17.32 ± 12.39% and enlarged the LVs (*R* = 0.705, *P* = .003) (Figure 2B). The association between the calculated %HSE and the GNS scores at 72 hours of survival was not significant (*R* = 0.235, *P* = 0.398) (Figure 2C). This suggests that the neurological deficit associated with AIS is not solely attributed to the progression of edema.

### Lesion volumes corrected for edema (LVc) display no relationship with GNS

**Figure 3.**
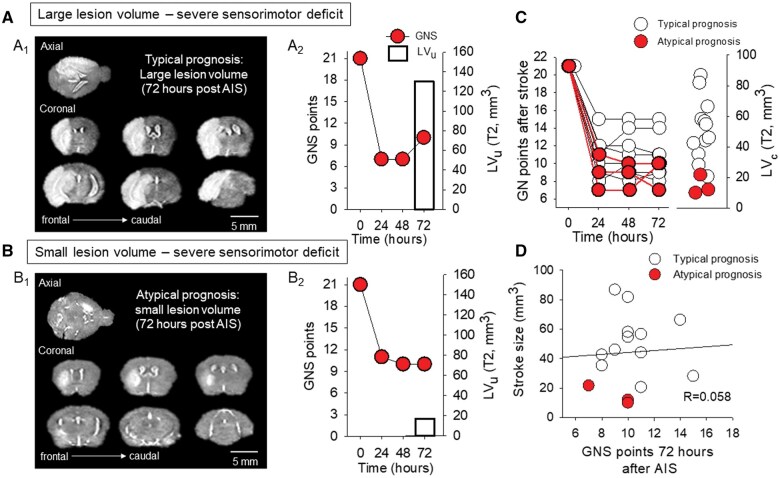
A subgroup of mice exhibited a paradoxical relationship between small lesion volume and severe neurological deficit. (A) A representative mouse with a typical prognosis displays a low Garcia Neuroscore Scale (GNS) score over the course of 72 hours of survival (A2), and a large lesion volume as measured on the T2-weighted MRI sequence (A1). (B) A mouse with severe neurological deficit (B2) was identified as having a poor prognosis and a small T2 lesion volume (B1). (C) Garcia Neuroscore Scale score assessed every 24 hours after AIS induction and corrected lesion volume (LVc) on day 3 are unrelated. (D) There was no linear relationship between lesion volume and GNS score assessed 72 hours after AIS (linear regression: *R* = 0.058, *P* = .837).

To accurately characterize the lesions, we corrected the LVs for edema (LVc) using a previously described formula.^27^ The application of edema correction resulted in a significant decrease in LVs (44.36 ± 23.56 vs 87.13 ± 30.64 mm^3^; LVc vs LV) (Figure 2D). However, no correlation was observed between LVs and GNS, either before or after edema correction (LVc) at the time of MRI (*R* = 0.0581, *P* = .837 and *R* = 0.167, *P* = .552; LVc and LV). The lack of correlation between the T2-weighted LVc and the GNS points at 24 (*R* = 0.170, *P* = .545), 48 (*R* = 0.0808, *P* = .775), or 72 hours of survival of the 15 mice (as shown above) confirms that the LVcs do not predict neurological outcome (Figure 3C and D). Furthermore, the volume of decreased ADC within T2 lesions did not correspond to the GNS score (*R* = 0.392, *P* = .149), indicating that the GNS score is not a reliable indicator of ADC volume. However, the GNS score measured at 24 hours strongly correlated with the GNS score at 72 hours of survival of the same animal (*R* = 0.748, *P* = .0013). Therefore, neurological deficits assessed at 24 hours of survival proved to be predictive of the GNS score at later time points.

The animals were categorized again based on LVc. Lesions <30 mm^3^ were classified as mild, those between 30 and 60 mm^3^ as moderate, and those larger than 60 mm^3^ as severe lesions (Figure S2). In line with the distribution of LVc, GNS scores did not differ between the groups at any investigated time point (*f*_group_ = 0.099, *p*_group_ = 0.907) and did not change significantly within groups over time (*f*_time_ = 0.983, *p*_time_ = 0.345).

### Small LVs are sometimes associated with severe neurological impairment

Three of the 15 mice evaluated exhibited a paradoxical relationship between LVs and neurological deficits, identified as “atypical prognoses” (Figure 3C). These animals, who formed the mild lesion group, as defined above, showed lower LVc compared to the rest of the mice (14.64 ± 6.25 vs 51.79 ± 19.96 mm^3^, atypical vs typical prognosis), but still showed severe residual deficits (GNS score at 72 h: 9 ± 2 vs 11 ± 2; atypical vs typical prognosis) (Figure 3C). MRI and GNS data for one mouse with an atypical prognosis are presented in Figure 3B1-2. Despite the presence of a mild lesion, a severe neurological deficit was observed. In contrast, the T2-weighted LVs were compatible with the measured neurological deficit in 12 out of 15 mice, identified as having a “typical prognosis”. For instance, the mouse presented in Figure 3A1-2 showed a typical prognosis due to the severe LV on the T2-weighted MRI sequences, which was accompanied by a severe neurological deficit represented by the low GNS score. The linear relationship between LVc and the GNS score weakened by day 3 of survival (*R* = 0.146, *P* = 0.61 vs *R* = 0.485, *P* = 0.110; 72 vs 24 hours) in the group of animals with typical prognosis. This finding suggests that the paradoxical relationship of the parameters is not caused by atypical cases. Finally, as expected, the corrected LV displayed no relationship with the GNS scores measured 72 hours after AIS (*R* = 0.058, *P* = 0.837). Importantly, the exclusion of “atypical cases” (*n* = 3 animals) had no significant effect on the data (*R* = 0.298, *P* = 0.92), as there was still no linear relationship between GNS scores and lesion volume in “typical cases.”

### Cortical LVs are associated with neurological impairment

**Figure 4.**
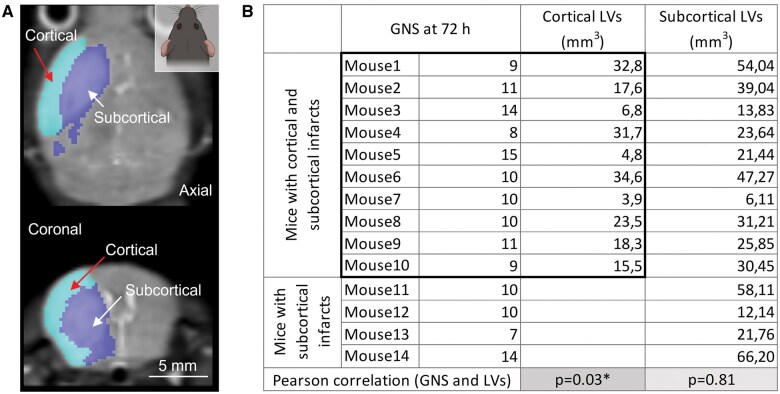
Cortical LVs show a linear relationship with neurological impairment 72 hours after stroke. (A) A representative axial (top) and coronal (bottom) T2 weighted sequence showing the semi-automated separation of cortical and subcortical infarcts. Light blue selection indicates cortical (red arrow), deep blue selection denotes subcortical (white arrow) infarcts. (B) The table shows the 2 subgroups of mice: (1) mice with cortical and subcortical LVs, and (2) mice with only subcortical LVs. Cortical LVs of mice with cortical and subcortical infarcts and the corresponding GNS scores at 72 hours are highlighted with a black rectangle. There was a linear relationship between cortical but not subcortical LVs and GNS scores assessed 72 hours after AIS (linear regression: *R* = 0.681, *P* = .03*).

To discriminate infarcted brain regions according to anatomical location, we relied on a semi-automated region of interest placement within the infarct area (Figure 4A). Based on the anatomical location of the infarcts, the animals were divided into 2 subgroups: (1) mice with subcortical and cortical infarcts (*n* = 10, Figure 4A), and (2) mice with subcortical infarcts only (*n* = 4). Interestingly, while subcortical LVs showed no relationship (*R* = 0.068, *P* = 0.81), cortical LVs showed a significant correlation with GNS scores measured 72 hours after AIS (*R* = 0.681, *P* = 0.03). These results suggest that the paradoxical relationship between LVs and GNS scores in the above-mentioned “atypical cases” may be due to the large variation in subcortical LVs (6.11-66.2 mm^3^) (Figure 4B).

## DISCUSSION

### The paradoxical relationship between infarct size and neurological deficit in the mouse MCAO model

This study provides evidence that infarct size is not a consistent indicator of sensorimotor deficits in acute ischemic stroke. In addition to some clinical reports, we have demonstrated here that there are unpredictable individual cases in the mouse MCAO model in which the expected trends do not prevail and infarct size does not show a correlation with the neurological outcome. The data presented here demonstrate that relying on lesion volume alone to predict AIS outcome may lead to an incorrect prognosis. In our experimental groups, one-fifth of the animals (3/15 mice) showed an “atypical prognosis” (mild lesion volume associated with severe neurological deficit). Exclusion or outlier filtering of these cases from experimental studies leads to incorrect conclusions because the data are overly optimized for homogeneity, do not represent variations in real populations, and thus do not translate to the clinical cases. Such irreproducible experimental AIS results have misled the field and contributed to the translational gap.^17^ Accordingly, further analysis of the results led to the observation that the location of the infarcts significantly influences the interpretation of the data. We found that while cortical LV showed a linear relationship, subcortical LV did not correlate with associated neurological deficits.

Stroke research is saturated with over 1000 successful preclinical pharmacological treatment studies that have failed to translate into routine clinical care.^17^ The reason for the continuous failure remains unclear, but several factors are suspected. For instance, in animal AIS models, the follow-up period is much shorter than in the clinic (typically between 1 week and 1 month), and there is no universal, widely accepted symptom scale, so lesion volume is taken as a standardized measure of outcome.^29^ Predicting functional outcome in rodents is as complicated and multifactorial as it is in humans. Furthermore, the intention to create standardized and uniform experimental groups for better statistical comparison may obscure the natural diversity of stroke presentation, which is found in humans.^30^

**Figure 5.**
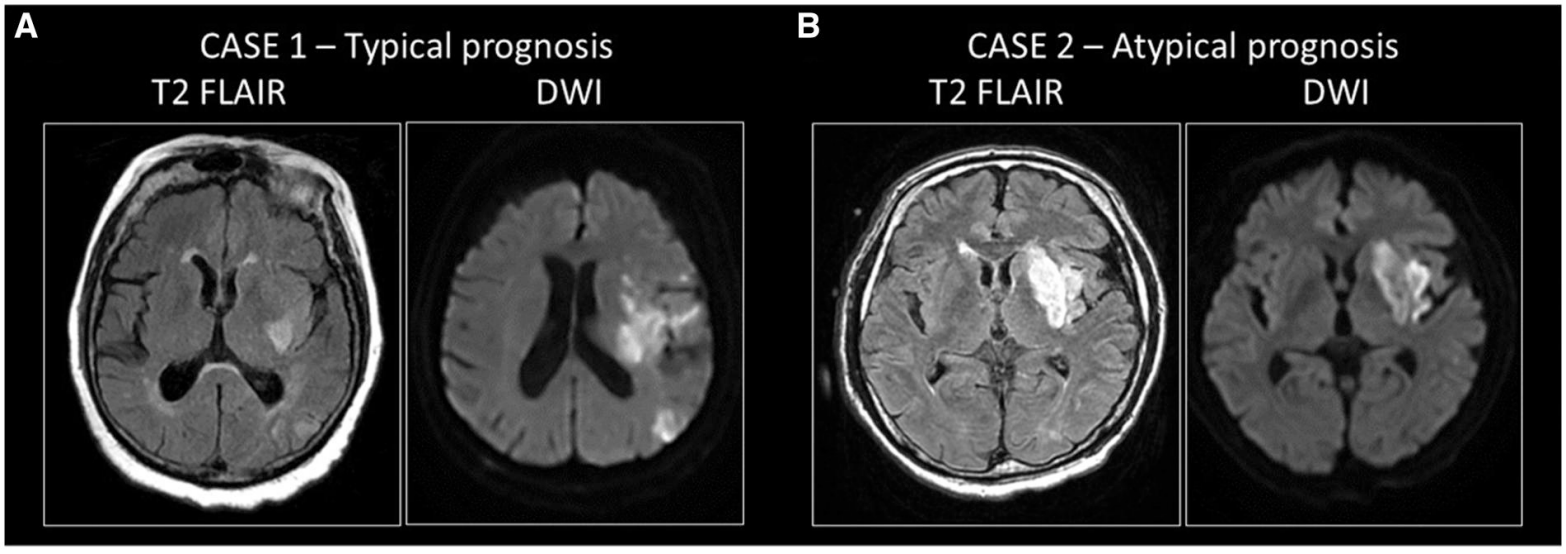
Clinical cases of typical and atypical prognosis in patients with ischemic stroke. (A) Case 1: typical prognosis in a patient with a large infarct volume with severe residual symptoms. An 81-year-old female patient was admitted with aphasia and severe right-sided hemiparesis. The National Institutes of Health Stroke Scale score at the initial examination was 14 points. The Alberta Stroke Programme Early CT score was 9/10. CT angiogram showed occlusion of the left middle cerebral artery (M1). The onset of symptoms was unknown. According to protocol, CT perfusion was performed, showing a large salvageable penumbra without core. Despite the favorable initial imaging results, after successful clot removal (eTICI 3), control MRI showed the development of a large infarct on both diffusion-weighted imaging (DWI) and FLAIR. National Institutes of Health Stroke Scale score at discharge was 22 points. The 90-day modified Rankin Scale (mRS) score, which is based on disability and dependence on activities of daily living following the stroke, was 5; the patient had severe residual symptoms. (B) Case 2: atypical prognosis in a patient with moderate infarct volume and severe residual symptoms. A 65-year-old male patient presented with aphasia and right-sided hemiparesis. The NIHSS at admission was 9 points. Alberta Stroke Programme Early CT Score score was 8/10. A CT angiogram showed occlusion of the left middle cerebral artery (M2). The onset of symptoms was unknown. According to protocol, CT perfusion was performed, showing a 54 ml penumbra and a small 4 ml core. Endovascular treatment was indicated and successfully performed (eTICI 3). Severe clinical progression was noted, with NIHSS 16 points at discharge, and a 90-day mRS of 5 points. The clinical data presented here were not collected specifically for this project. These patient data were selected for representative purposes for this article and were obtained from the database of the Department of Neurology, Albert Szent-Györgyi Health Centre, University of Szeged, under the supervision of Dr Nikoletta Szabó.

The variability of infarct size in rodent MCAO models complicates and biases the desired homogeneity of subjects.^17^ Although robust earlier studies demonstrated significant correlations between infarct volumes and neurological outcomes in mice and rats,^16^ the predictive value of early infarct assessment, such as neuroimaging at 24 hours, has been shown to depend on the timing of MRI and the duration of MCAO.^31^ A more recent study confirms that lesion topology may increase predictive accuracy when using neuroimaging to characterize residual deficit in mice.^31^ Importantly, the same study suggests that a subset of anatomical structures within the infarct area may be particularly influential in predicting long-term stroke outcome. Taking this into account, 2 of the possible explanations for the atypical prognosis in our study, among many others, could be (i) the localization of the infarct,^32^^,^^33^ whose inclusion in the prediction model could also improve the estimation of residual deficit^31^ or (ii) the clinical-DWI mismatch phenomenon known from clinical studies.^34–36^ The clinical-DWI mismatch describes that severe acute clinical symptoms may be accompanied by mild visible LV on DWI images, which compromises the evaluation or prediction of early AIS outcome and final lesion size.^37^ In the subacute phase, discrepancy between imaging results and neurological status may signal lesion evolution by a secondary pathological mechanism. Also, clinically observed severe symptoms inconsistent with small LV may result from dynamic collateral recruitment of distant brain regions.^38^ Consistent with this, AIS patients with perfusion deficits have higher NIHSS scores.^13^^,^^38^ Taken together, the paradoxical relationship of sensorimotor deficit and lesion volume after AIS is also detectable in clinical settings (Figure 5), and this paradox may be resolved by careful assessment of cortical involvement (Figure 4).

### The role of edema progression in the mouse AIS model

The development of ischemic lesions is always associated with cerebral edema. The swelling is more pronounced in patients with successful recanalization because the restoration of active blood flow is accompanied by ion and water entry through the damaged capillary walls into the brain.^39^ The development of brain edema typically peaks on day 3-4 and has been shown to increase the mortality rate of MCA territory strokes by 80%, as the initial swelling progresses to space-occupying edema.^40^ The most severe form of brain edema following AIS involving the entire MCA territory is called malignant MCA infarction, which is known to have devastating effects on patients because of the compression of surrounding tissue, possible herniation, and death in one-fifth of patients within the first week.^41^ Cerebral edema is now the subject of intense research in animal models.^42^^,^^43^ Since focal brain edema exerts compression on the surrounding tissue and expands the size of the lesion, the volume of edema, and the volume of the edema-free infarcted regions can be calculated for accurate tracking of infarct size. We chose to use edema correction according to a reproducible method used to estimate brain swelling in rodents.^27^ Although large lesional swelling volume has been found to predict poor outcome in patients with hemispheric stroke, edema alone does not account for the deficits observed in our study.^44^ However, edema progression greatly masked LV and hindered the accurate estimation of stroke size in our model.

### Limitations

Among the limitations of this study, we note that the GNS scoring in mice is obviously limited to mainly sensorimotor tasks, gait and balance functions, whereas the clinical routine NIHSS is a more comprehensive scale that also assesses visual and language status of patients. For this reason, the assessment of neurological deficits in mice may lack some clinically relevant, patient-feedback-controlled tasks that critically influence the interpretation of the data. The other issue is the short follow-up period after AIS in our and other rodent AIS models. Our study period covered only the subacute phase of up to 72 hours post-AIS, which is incomparably shorter than the gold standard 90-day post-AIS control measurement of neurological status in patients. In fact, initial modified Rankin Scale and NIHSS scores have been shown not to correlate with the outcome of AIS patients at 90 days post-AIS.

Although more detailed histopathological analysis would improve the quality of these results, the spatial resolution of the MEDISO 3 T rodent MRI system used was a limiting factor for reproducible anatomical analysis. In order not to compromise the validity and reproducibility of our observations, we relied on cortical and subcortical separation of the data.

## CONCLUSIONS

This study provides valuable insights into the complex relationship between LV and neurological deficits in AIS models. Based on our data, we suggest that, to improve the reproducibility of rodent AIS models, future experimental studies should be conducted with increased numbers of animals, longer follow-up, a more comprehensive functional test battery, and minimal exclusion, filtering, and normalization of outliers.

## Supplementary Material

nlaf046_Supplementary_Data

## Data Availability

The data that support the findings of this study are available on request from the corresponding author. The data (ie, raw DICOM mouse MRI sequences, raw DICOM human MRI sequences, tables with mouse GNS scores) are archived on our dedicated Sinology (NAS server) platform and are not publicly available due to privacy or ethical restrictions.
