# Supplementary material for: The paradoxical relationship of sensorimotor deficit and lesion volume in acute ischemic stroke
Source: J Neuropathol Exp Neurol. 2025 Apr 24;84(9):771–9. doi: 10.1093/jnen/nlaf046 (PMC12365491; doi:10.1093/jnen/nlaf046)
Supplement: nlaf046_Supplementary_Data [file nlaf046_supplementary_data.zip › Supplementary_grouping of animals.pptx]

## Slide 1
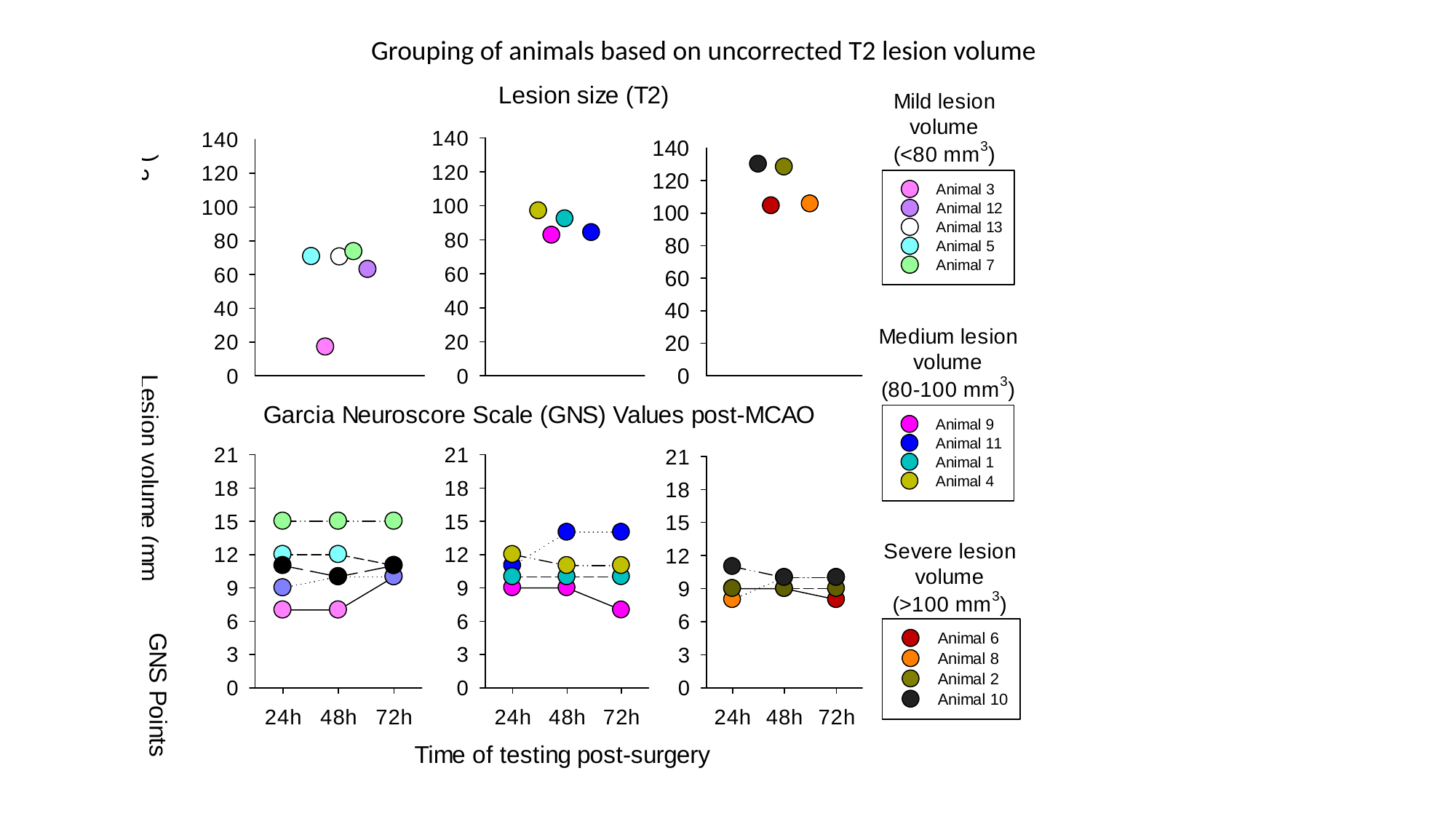

Grouping of animals based on uncorrected T2 lesion volume

## Slide 2
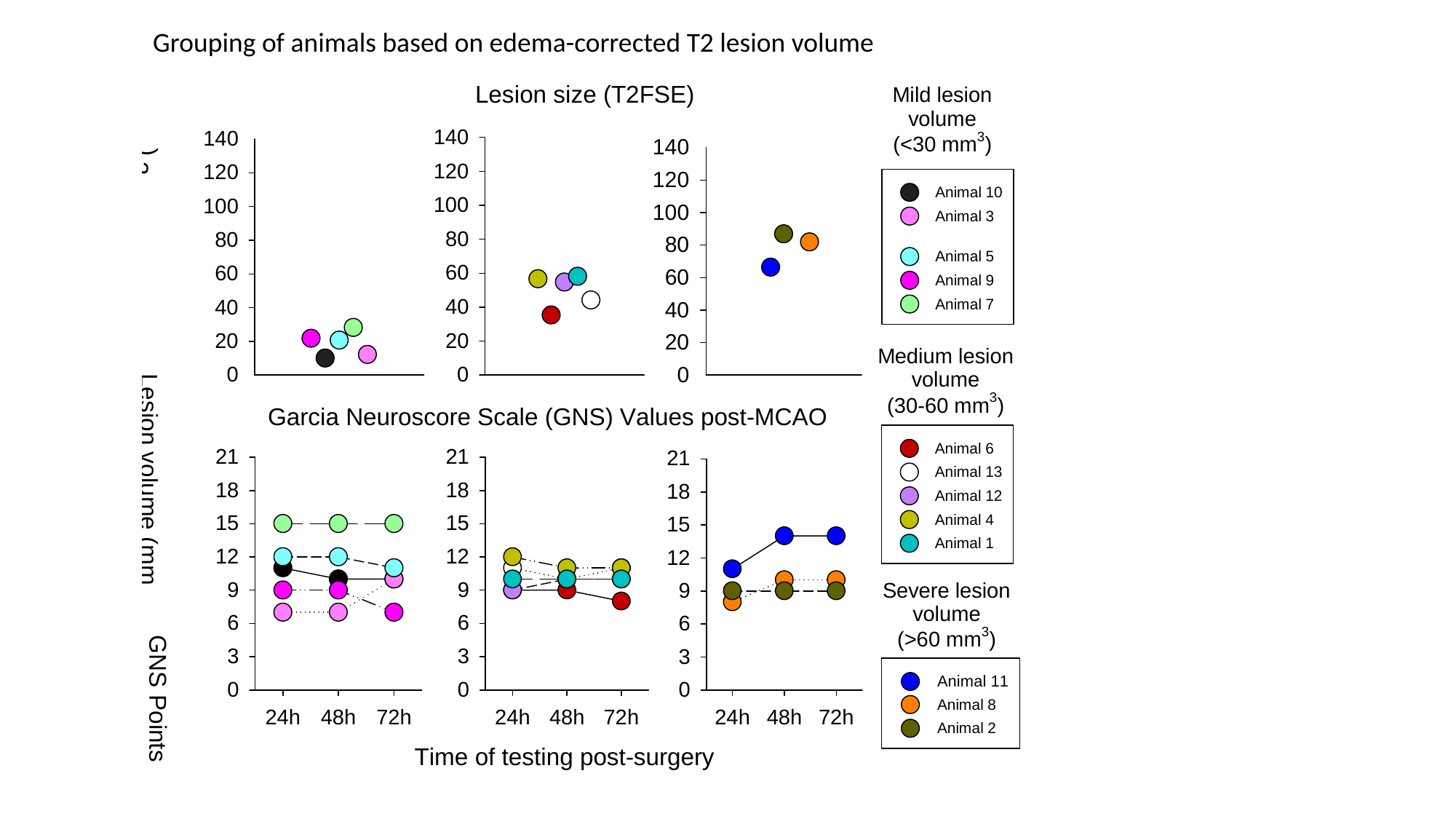

Grouping of animals based on edema-corrected T2 lesion volume
